# Beneficial effects on T cells by photodynamic therapy with talaporfin enhance cancer immunotherapy

**DOI:** 10.1093/intimm/dxaf003

**Published:** 2025-01-22

**Authors:** Ehab M Ezzaldeen, Tomonori Yaguchi, Ryotaro Imagawa, Mohamed A Soltan, Akira Hirata, Kosaku Murakami, Hirotake Tsukamoto, Manabu Muto, Tasuku Honjo, Kenji Chamoto

**Affiliations:** Department of Immunology and Genomic Medicine, Center for Cancer Immunotherapy and Immunobiology, Kyoto University Graduate School of Medicine, Kyoto, Japan; Department of Immunology and Genomic Medicine, Center for Cancer Immunotherapy and Immunobiology, Kyoto University Graduate School of Medicine, Kyoto, Japan; Department of Immuno-Oncology PDT, Kyoto University Graduate School of Medicine, Kyoto, Japan; Department of Immunology and Genomic Medicine, Center for Cancer Immunotherapy and Immunobiology, Kyoto University Graduate School of Medicine, Kyoto, Japan; Department of Immunology and Genomic Medicine, Center for Cancer Immunotherapy and Immunobiology, Kyoto University Graduate School of Medicine, Kyoto, Japan; Pharmacokinetics and Toxicology Group, Drug Discovery Department, R&D Division, Meiji Seika Pharma Co., Ltd, Tokyo, Japan; Division of Clinical Immunology and Cancer Immunotherapy, Center for Cancer Immunotherapy and Immunobiology, Kyoto University Graduate School of Medicine, Kyoto, Japan; Division of Clinical Immunology and Cancer Immunotherapy, Center for Cancer Immunotherapy and Immunobiology, Kyoto University Graduate School of Medicine, Kyoto, Japan; Department of Medical Oncology, Kyoto University Graduate School of Medicine, Kyoto, Japan; Department of Immunology and Genomic Medicine, Center for Cancer Immunotherapy and Immunobiology, Kyoto University Graduate School of Medicine, Kyoto, Japan; Department of Immunology and Genomic Medicine, Center for Cancer Immunotherapy and Immunobiology, Kyoto University Graduate School of Medicine, Kyoto, Japan; Department of Immuno-Oncology PDT, Kyoto University Graduate School of Medicine, Kyoto, Japan

**Keywords:** abscopal effect, ferroptosis, immune checkpoint blockade

## Abstract

Photodynamic therapy (PDT), a local cancer treatment using photosensitizers, has been reported to enhance antitumor immune responses by inducing immunogenic cell death. Although several studies have demonstrated the synergistic antitumor effects of PDT and immune checkpoint blockage (ICB), the detailed underlying mechanisms remain poorly understood. In this study, we investigated the immunological effects of PDT with talaporfin (Tal-PDT), a clinically approved photosensitizer, using bilateral tumor-bearing mouse models. Treatment with Tal-PDT on the tumor on one side of the mouse resulted in tumor growth inhibition on the untreated opposite side. This phenomenon, accompanied by tumor antigen-specific immune reactions, is indicative of an abscopal effect. When combined with anti PD-L1 antibody, synergistic antitumor effects were observed on both the laser-treated and untreated sides. Mechanistically, Tal-PDT enhanced the induction of XCR-1^+^ dendritic cells in the proximal draining lymph node likely through the induction of ferroptosis in tumor cells. This, in turn, led to the systemic generation of precursor-exhausted CD8^+^ T cells. Moreover, talaporfin was selectively incorporated into tumor cells rather than into tumor-infiltrating T cells *in vivo*, leading to targeted tumor killing while preserving T cells. These beneficial effects of Tal-PDT on antitumor immunity collectively enhance ICB cancer immunotherapy. Our study demonstrates the potential of combining Tal-PDT with ICB therapy for clinical applications.

## Teaser

Mechanisms for the abscopal anti-tumor effects of photodynamic therapy

## Introduction

Cancer treatment has been transformed by the emergence of immunotherapy, particularly immune checkpoint blockade (ICB) therapies like programmed cell death 1 (PD-1) inhibitors. While these therapies have shown impressive results, they still face significant challenges, including the development of resistance (1). One major resistance mechanism is defective T-cell priming in draining lymph nodes (dLNs), caused by an insufficient number and inadequate presentation of cancer antigens by antigen-presenting cells (2, 3). Local therapies like radiotherapy and certain chemotherapeutic drugs may overcome this resistance by inducing immunogenic cell death (ICD). ICD releases cancer antigens and damage-associated molecular patterns (DAMPs), which activate antigen-presenting cells (4). In animal models and some human clinical trials, these therapies have shown synergistic effects when combined with ICB (5). However, their therapeutic potential is limited by adverse consequences, such as immune cell cytotoxicity (6).

Photodynamic therapy (PDT) is an effective local cancer therapy. It induces an antitumor effect with minimal side effects, unlike methods such as radiotherapy (7, 8). PDT involves the systemic injection of a photosensitizer, which selectively accumulates in tumor cells (9, 10). Exposure to specific wavelengths of light activates the photosensitizer, leading to localized killing of cancer cells primarily through the generation of reactive oxygen species (ROS) (11). Since PDT can trigger ICD (12), it may be a promising candidate for combination therapies with ICB. Several studies have reported synergistic effects of these combination therapies in preclinical models (13). While many photosensitizers are in (pre)clinical trials, only a few received clinical approval (14). Talaporfin is one of these approved drugs and is used in Japan for treating esophageal cancer and lung cancers. To effectively apply the PDT and ICB combination therapy in the clinic, it is essential to analyze the immunologic effects of PDT using talaporfin (Tal-PDT).

PDT induces both immunogenic and non-immunogenic cell death. Ferroptosis is a form of regulated necrotic cell death associated with iron-dependent oxidative modification of phospholipid membranes (15). Ferroptosis has gained increasing attention in PDT because PDT generates ROS and lipid peroxides, which are crucial for triggering ferroptosis (16). Although several studies have reported that ferroptosis can be immunogenic, its immunogenicity is highly context-dependent (17). Tal-PDT has been reported to induce ferroptosis in cancer cells (16), but its immunogenicity has not yet been evaluated.

Similar to other photosensitizers, talaporfin has been reported to enhance the therapeutic efficacy of PD-1 inhibitors. In a bilateral mouse cancer model, where cancer cells were inoculated on both sides of the mice, PDT exposure on one side exerted synergistic antitumor effects not only on the laser-treated side but also on the untreated side. This phenomenon is known as the abscopal effect (18). The detailed immunological mechanism by which Tal-PDT therapy induces the abscopal effect has not been elucidated.

In this study, we assessed the immunological effects of Tal-PDT using bilateral tumor-bearing mouse models. We found that ferroptosis induced by Tal-PDT in cancer cells and the proximal dLN was essential for the observed abscopal effects. Combining Tal-PDT with PD-1 inhibitors showed synergistic abscopal effects, with activation of conventional type I dendritic cells (cDC1s) in the proximal dLN and generation of replicable precursor-exhausted T cells (Tpex cells) in the distal tumor sites. Importantly, talaporfin was selectively incorporated into tumor cells rather than tumor-infiltrating T cells, thereby mitigating potential Tal-PDT-induced damage to T cells. These immunologic effects of Tal-PDT are beneficial for anti-tumor immunity and collectively induce systemic anti-tumor immune activity, which is an abscopal effect. Our study provides proof of concept for the combined use of Tal-PDT and PD-1 blockade therapies in clinical settings.

## Methods

### Regents and antibodies

All reagents and antibodies used are listed in Supplementary Tables 1 and 2.

### Animal experiments

Male C57BL/6J and BALB/c mice (aged 7–12 weeks) were obtained from SLC and housed under specific pathogen-free (SPF) conditions. OT-1 TCR-transgenic mice were purchased from The Jackson Laboratory (originally from M. B. Bevan at University of Washington). All mice were handled under protocols approved by the respective animal care and use committee. For all tumor cell lines used, 5 × 10^5^ cells were intradermally inoculated into the right thigh (laser-irradiated site), and 2.5 × 10^5^ cells were inoculated into the left flank site (untreated site). Anti-PD-L1 monoclonal antibody (mAb) (clone 1-111A.4) was produced as previously described (19). This antibody was intraperitoneally (i.p.) injected starting on Day 7, with injections every 6 days in the MC38-bearing mice (1 mg/kg) or every 4 days in the CT26-bearing mice (2.5 mg/kg). An isotype (rat IgG2a, κ) control antibody (Bio X Cell, Lebanon, NH, USA) was injected in the control mice. Liproxstatin-1 (Merck, Darmstadt, Germany) dissolved in DMSO was i.p. injected at a dose of 20 mg/kg daily for 7 days starting on Day 6. Tumor volume was calculated using the following formula: (length) × (width) × (height) × 3.14/6.

### Cell lines

MC38 and CT26 cells were obtained from the Kerafast Cell Bank and the National Cancer Institute, respectively. BPmel-1, a murine melanoma cell line with a BRAF-mutated/PTEN-loss genotype, was established from a Cg-*Braf*^*tm1Mmcm*^*Ptent*^*m1Hwu*^ Tg (Tyr-cre/ERT2) 13Bos/BosJ mouse (20) as previously described (21). To overexpress the artificial antigen SIY (SIYRYYGL), three copies of the SIY minigene, conjugated with a spacer sequence (SIYRYYGL-AAY) (22), were transduced into BPmel-1 using third-generation HIV vectors (21, 23). These vectors included a puromycin resistance gene under the control of the internal ribosomal entry site (IRES). BPmel-1 cells overexpressing SIY (BPmel-1-SIY) were selected using puromycin (Thermo Fisher Scientific, Waltham, MA, USA) (2500 ng/ml). CT26 and BPmel-1-SIY cells were cultured in RPMI 1640 media. MC38 cells were cultured in DMEM media. The media were supplemented with 10% (v/v) heat-inactivated fetal calf serum (FCS) and a mixed solution of 1% (v/v) penicillin‒streptomycin (Nacalai Tesque, Kyoto, Japan). For the evaluation of PD-L1 expression, MC38 and CT26 were treated with mouse IFN-γ (50ng/ml) (Thermo Fisher Scientific) for 24h.

### PDT

Talaporfin (Meiji Seika Pharma, Tokyo, Japan) dissolved in PBS was *i.v.* administered via the retro-orbital sinus in mice (7.5 mg/kg), performed in a dark room. Two hours after the talaporfin injection, laser therapy was applied to the proximal tumor at the dose of 100 J/cm^2^ (irradiance: 150 mW/cm^2^ × 667 seconds) using a semiconductor laser device (wavelength; 664 nm ± 2 nm, maximum output power; 600 mW) (Meiji Seika Pharma). Mice were anesthetized with isoflurane during the procedure and were kept in the dark room for 24 hours after PDT.

### Tissue processing

Upon euthanasia with CO_2_, in accordance with the ethical protocol, the tumors were excised, weighed, and minced into small 2 mm pieces using scissors. The tissue was digested using collagenase type 4 (1 mg/ml) (Worthington Biochemical Corporation, Lakewood, NJ, USA) in 3 ml of plain RPMI using a gentleMACS™ Octo Dissociator (Miltenyi Biotec, Bergisch Gladbach, Germany) for approximately 42 minutes. Then, 7 ml of 10% FCS RPMI complete media was added to neutralize the collagenase. The digested tumor was passed through a 70 µm filter to obtain a single-cell suspension. The volume was adjusted to 20 mg/100 µl, corresponding to approximately 1 × 10^6^ cells in 100 µl. For the dLNs, the inguinal and axillary LNs near the tumor were harvested. The dLNs were ground with a syringe plunger and incubated in a 37°C CO_2_ incubator for 30 minutes with plain RPMI supplemented with 3 ml of collagenase type 4 (1 mg/ml) by pipetting twice every 15 minutes. Then, the suspension was passed through a 70 µm filter, and collagenase activity was neutralized by adding 10% FCS RPMI complete media (7 ml). The cell count was adjusted to 1 × 10^6^ cells/100 µl.

### Flow cytometry analysis

After processing the tumor and dLN samples, 1 × 10^6^ cells from each single-cell suspension were seeded into a 96-well V-shaped plate. The Fc receptors on cells were blocked using 100 µl (5 µg/ml) of anti-mouse CD16/32 antibody for 20 minutes at 4°C. Dead cells were excluded using Zombie NIR Live/Dead staining. To stain the cell surface, a master mix of the indicated antibodies was prepared by diluting in Brilliant Stain buffer and FACS solution (2% FCS in PBS) mixed in a 3:1 ratio. Dendritic cells (DCs) were defined as CD45^+^, Gr-1^−^, F4/80^−^, CD11c^+^. Tpex cells were defined as CD45^+^, CD8^+^, CD44^+^, PD-1^int^, TCF-1^+^, Tim-3^−^ cells. One hundred microliters of the mixture were added to each sample and incubated in the dark for 30 minutes at 4°C. Then, the cells were washed twice with 200 µL of FACS solution. To assess SIY-specific CD8^+^ T cells, the cells were stained with SIY-tetramer (MBL, Tokyo, Japan) for 30 minutes at 4°C. β-gal-tetramer (MBL) was used as a negative control. After staining, the cells were fixed using the Foxp3/Transcription Factor Fixation/Permeabilization Concentrate and Diluent kit (Thermo Fisher Scientific) according to the manufacturer’s instructions. Following staining, samples were subjected to flow cytometry analysis. For intracellular staining, cells were processed with Foxp3/Transcription Factor Fixation/Permeabilization Concentrate and Diluent kit (Thermo Fisher Scientific) according to the manufacturer’s instructions. The absolute count of the cells was calculated using CountBright Absolute Counting Beads (Thermo Fisher Scientific). Data were collected using an LSR Fortessa or Symphony A5 flow cytometer (Becton, Dickinson and Company) and analyzed using FlowJo software version 10.8.1 (Becton, Dickinson and Company).

### PDT and cell viability assessment

For Liproxstatin-1 treatment, MC38 cells were cultured with 10 μM Liproxstatin-1 for 48 hours. The cells were then washed and treated with 15.6 μM talaporfin for 5 hours under light-protected conditions. The cells were then washed again and irradiated with the PDT laser at a dose of 7.5 J/cm^2^ (irradiance: 25 mW/cm^2^ × 300 seconds). Following irradiation, the cells were cultured for 24 hours. The viability of each cell was assessed by Zombie NIR (Biolegend, San Diego, CA, USA) and Apotracker Green (Biolegend) staining. The cells were analyzed using Symphony A5 flow cytometer (Becton, Dickinson and Company). For the comparison experiment between MC38 and T cells, freshly isolated splenocytes and MC38 cells were incubated with 5 μM talaporfin for 2 hours under light-protected conditions. The cells were then washed and irradiated with the PDT laser at a dose of 100 J/cm^2^ (irradiance: 150 mW/cm^2^ × 667 seconds). Following irradiation, the cells were cultured for 24 hours. The viability of each cell was assessed by FITC Annexin V Apoptosis Detection Kit with 7-AAD (Biolegend). The cells were analyzed using ID7000, a spectral cell analyzer (Sony, Tokyo, Japan).

### BODIPY staining and lipid peroxidation measurement

To assess lipid peroxidation in cells subjected to PDT with or without Lipro-1 or camptothecin treatment, MC38 cells (5 × 10^3^/well) were seeded in a 96-well black plate and incubated overnight. The cells were then treated with either 10 µM Lipro-1 for 48 hours or 10 µM camptothecin. Control cells were treated with DMSO. Following treatment, the cells were washed with PBS and incubated with 15.6 μM talaporfin for 5 hours. After washing twice with PBS, the cells were suspended in fresh media and subjected to laser irradiation at a dose of 7.5 J/cm^2^ (irradiance: 25 mW/cm^2^ × 300 seconds). The cells were then incubated at 37°C overnight, harvested with trypsinization, and stained with 5µM BODIPY 581/591 C11 dye (Thermo Fisher Scientific) (100 µl/well) dissolved in plain PBS for 20 minutes at 37°C. Flow cytometry analysis was performed to detect reduced and oxidized BODIPY C11 as PE-Texas Red and FITC fluorescence, respectively using Symphony A5 flow cytometer (Becton, Dickinson and Company). The data are expressed as the ratio of oxidized-to-nonoxidized mean fluorescence intensity (MFI).

### HMGB-1 ELISA

Mouse serum was collected from the cheek area and allowed to clot at room temperature for one hour without disturbance. The clots were removed by sequential centrifugation at 800 G for 15 minutes at 4°C. High mobility group box 1 (HMGB-1) levels in murine serum were quantified 48 hours after PDT treatment using the Mouse HMG1/HMGB1 ELISA Kit (LifeSpan Biosciences, Lynnwood, WA, USA).

### Talaporfin incorporation by T cells and MC38 tumor cells

To evaluate talaporfin incorporation *in vitro*, freshly isolated splenocytes, activated T cells which were generated from splenocytes stimulated with anti-CD3 Ab (10 µg/ml) and IL-2 (50 U/ml) for 3 days, or MC38 cells were incubated with 5 μM talaporfin for 2 hours under light-protected conditions. To evaluate talaporfin incorporation *in vivo*, talaporfin was administered intravenously to mice 8 days after MC38 inoculation. Tumor tissues were harvested 2 hours after administering the drug and digested with collagenase Type 4. Both the splenocytes (*in vitro*) and the digested tumor tissue (*in vivo*) were stained with CD4 (BUV563, GK1.5), CD8a (AF488, 53-6.7), CD45 (V500, 30-F11), TCR-b (BV650, H57-597), and analyzed using ID7000, a spectral cell analyzer (Sony). Single-color control spectra for each fluorochrome and talaporfin were acquired prior to analyzing experimental samples.

### Measurement of taraporfin concentration

The tumor tissue was homogenized with 49 volumes of homogenate buffer [10 mmol/l HEPES 50 mmol/l EDTA solution: methanol = 1:9 (v/v)]. Taraporfin concentrations in plasma and tissue homogenate were measured by using liquid chromatography combined with a tandem mass spectrometry (LC–MS/MS) system with positive-ion electrospray ionization (Nexera X2; SHIMADZU CORPORATION, Kyoto, Japan) (QTRAP 6500+; SCIEX, Framingham, MA, USA).

### Evaluation of the ability of DCs to stimulate T cells

On Day 14 after tumor inoculation, DCs were sorted from the proximal dLN of MC38 tumor-bearing mice (PDT-treated group and untreated control group) using CD11c MicroBeads (Miltenyi Biotec). These cells (1 × 10^4^/well) were co-cultured with naive OT-1 CD8⁺ T cells (5 × 10^4^/well), which had been isolated from the spleens of OT-1 mice and labeled with CFSE (Thermo Fisher Scientific) according to the manufacturer’s instructions, in a 96-well flat-bottom plate. The co-culture included OVA peptide (MBL) (0.5 µg/ml) and human IL-2 (Novartis, Basel, Switzerland) (20 IU/ml). After three days, proliferating OT-1 CD8⁺ T cells were assessed by CFSE dilution using flow cytometry.

### Statistical analyses

Statistical analyses were performed using Prism 8 (GraphPad Software, Boston, MA, USA). For multiple-group analysis, one-way or two-way analysis of variance (ANOVA) was used. To compare two groups, two-tailed unpaired Student’s *t* tests or Mann–Whitney tests were used.

## Results

### Local Tal-PDT induces the abscopal effect by enhancing infiltration of tumor-specific CD8^+^ T cells in laser-untreated distant tumors

**Figure 1.**
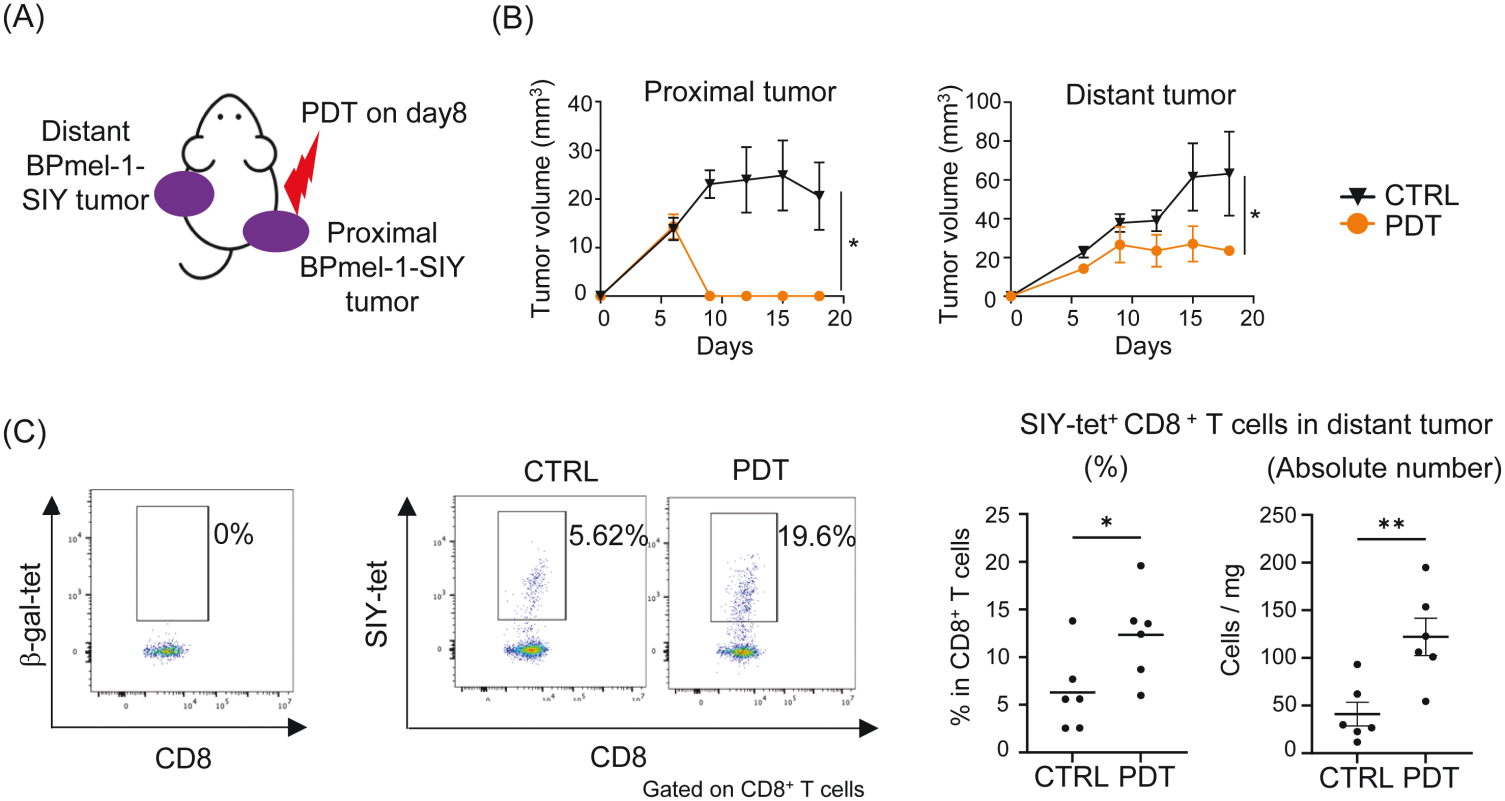
Local Tal-PDT therapy induces the abscopal effect, which is associated with increased infiltration of tumor antigen-specific CD8^+^ T cells into the laser-unexposed tumor. (A) Illustration of the experiment. (B) The growth of the proximal tumor (left panel) and distant tumor (right panel) (*n* = 3–5). (C) The SIY-specific CD8^+^ T cells in the distant tumor were analyzed on Day 14 by flow cytometry using the SIY-tetramer. β-gal tetramer was used as a negative control. Representative SIY-tetramer staining for each group is shown in the left panel. The frequency (middle panel) and the absolute number (right panel) of SIY-tet^+^ CD8^+^ T cells for all individuals are shown. Data are expressed as means ± SEM. Statistical analyses were performed using Mann–Whitney test (Day 18; B) and *t*-test (C). *P < .05, **P < .01. The data are representative of two independent experiments.

To understand how Tal-PDT induces the abscopal effect, we established a bilateral cancer model. In this model, a H-2K^b^-restricted model antigen, SIY, was overexpressed in a murine melanoma cell line, BPmel-1 (Fig. 1A) (21). We then performed PDT on the proximal tumor using talaporfin. We found that Tal-PDT not only suppressed the tumor at the treated site but also inhibited the growth of the laser-untreated distant tumor (Fig. 1B). Importantly, the number of SIY-specific CD8^+^ T cells, measured in a SIY-tetramer assay, increased in the distant tumor (Fig. 1C). This suggests that Tal-PDT induced the abscopal effect by enhancing tumor-specific T-cell responses in the laser-untreated distant tumor.

### Ferroptosis in laser-treated tumor cells is crucial for the abscopal effect of Tal-PDT

**Figure 2.**
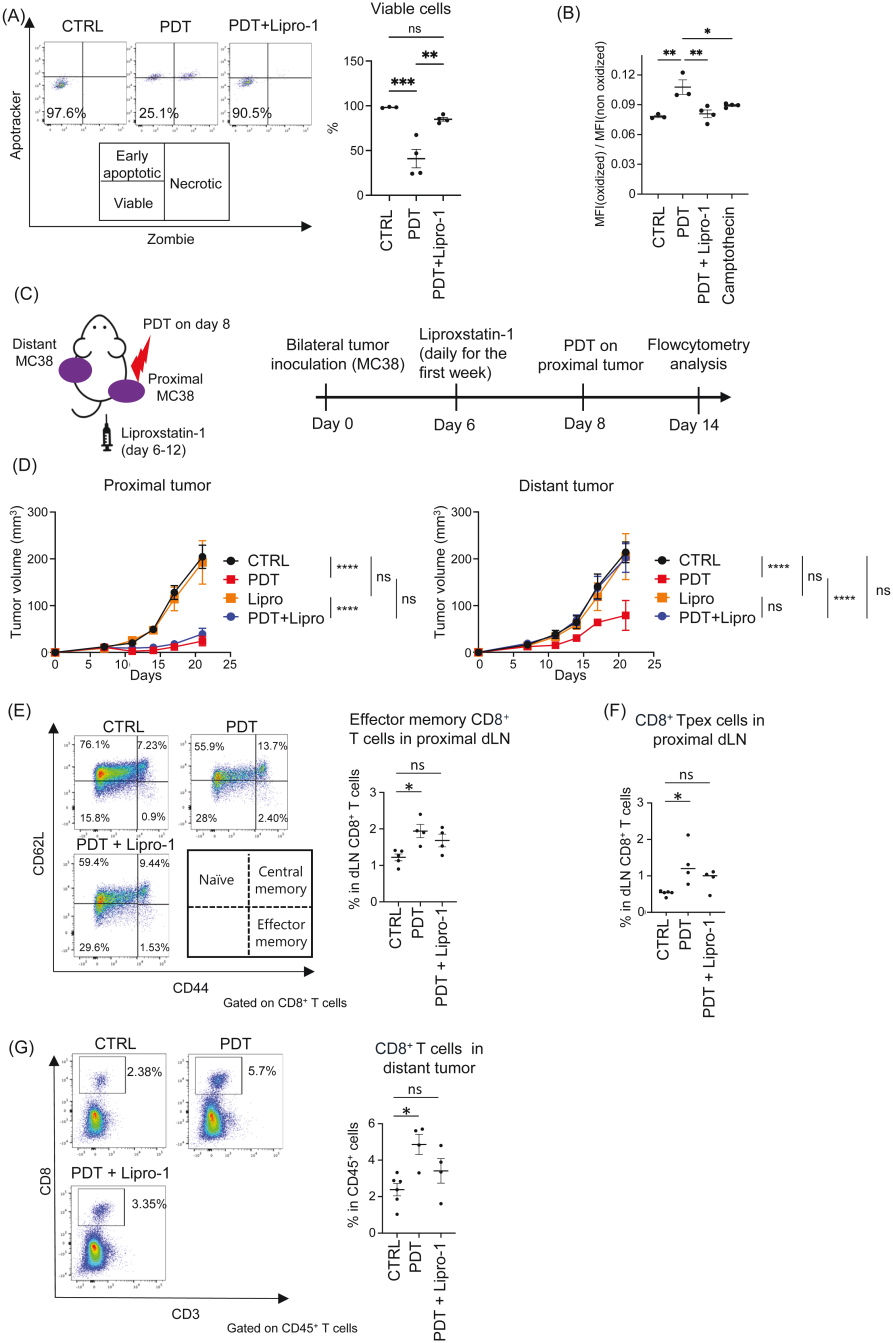
Tal-PDT-induced ferroptosis in tumor cells is essential for the effective abscopal effect. (A and B) MC38 cells were treated with liproxstatin-1 for 48 hours and then treated with talaporfin for 5 hours, after which the laser was emitted. Cell viability was evaluated 24 hours after laser irradiation. Representative staining in each group (left panel) and the percentage of viable cells for all individual wells (right panel) are shown (A). Lipid peroxidation was evaluated by flow cytometry using BODIPY581/591-C11. This probe detects non-oxidized lipids with PE Texas fluorescence and oxidized lipids with FITC fluorescence. The ratio of the mean fluorescence intensity (MFI) of FITC (oxidized) to MFI of PE Texas (non-oxidized) 16 hours after laser irradiation is shown. Camptothecin, an apoptosis inducer, was used as a negative control (B). (C-G) The role of ferroptosis in the abscopal effect was analyzed *in vivo*. Illustration of the experiment (C). Tumor growth of the proximal tumor (left panel) and the distant tumor (right panel) (*n* = 3–4) (D). The frequency of CD8^+^ effector memory (CD44^+^, CD62L^−^) T cells (E) and CD8^+^ Tpex cells (CD44^+^, PD-1^int^, TCF-1^+^, Tim-3^−^) (F) in the proximal dLN was assessed by flow cytometry. CD8^+^ T-cell infiltration in the distant tumor was assessed by flow cytometry (G). Representative flow cytometry staining results are shown in the left panels (E and G). Gating strategy of Tpex is shown in Supplementary Fig. 3. Data are expressed as means ± SEM. Statistical analyses were performed using one-way ANOVA followed by Tukey’s multiple comparisons test (A and B) or by Dunnett’s multiple comparisons test (E, F, and G) and two-way ANOVA followed by Tukey’s multiple comparisons test (Day 21; D) *P < .05, **P < .01, ***P < .001, ****P < .0001. ns; not significant. The data are representative of two independent experiments.

Ferroptosis is one of the immunogenic cell death modalities induced by PDT (12). Here, we investigated whether Tal-PDT-triggered ferroptosis of cancer cells is associated with the abscopal effect. The cytotoxicity by Tal-PDT on MC38 was attenuated *in vitro* in the presence of a ferroptosis inhibitor, liproxstatin-1 (Fig. 2A). We also evaluated one of the ferroptosis markers, lipid peroxidation, using BODIBY581/591-C11 staining. We found that PDT substantially induced lipid peroxidation compared with camptothecin, an apoptosis inducer, while treatment with liproxstatin-1 prevented the upregulation of lipid peroxidation (Fig. 2B). These results suggest that cell death was mediated by lipid peroxidation-dependent ferroptosis in this therapy. To evaluate the role of ferroptosis in the abscopal effect *in vivo*, proximal tumors were treated with Tal-PDT on day 8, and liproxstatin-1 was administered from Day 6 to Day 12 (Fig. 2C). While the direct effect of Tal-PDT on proximal tumors was still significantly observed, probably due to types of cell death other than ferroptosis (12, 24), the abscopal effect on distant tumors was largely negated by liproxstatin-1 treatment (Fig. 2D). Importantly, liproxstatin-1 treatment also abolished the following immunological effects of Tal-PDT. In the proximal dLN, the increase in the number of effector memory CD8^+^ T cells and CD8^+^ Tpex cells (CD44^+^, PD-1^int^, TCF-1^+^, Tim-3^−^), which are known to carry proliferative and long-survival capacity, was abolished (Fig. 2E and F). In the distant tumor tissue, the enhancement of T-cell infiltration was abolished (Fig. 2G). These results suggest that ferroptosis induced by Tal-PDT treatment is essential for immune activation in the dLN and the resulting abscopal effect.

### The combination of Tal-PDT and PD-1 inhibitors induces synergistic abscopal effects, characterized by increased XCR-1^+^ DCs and CD8^+^ precursor-exhausted T cells

**Figure 3.**
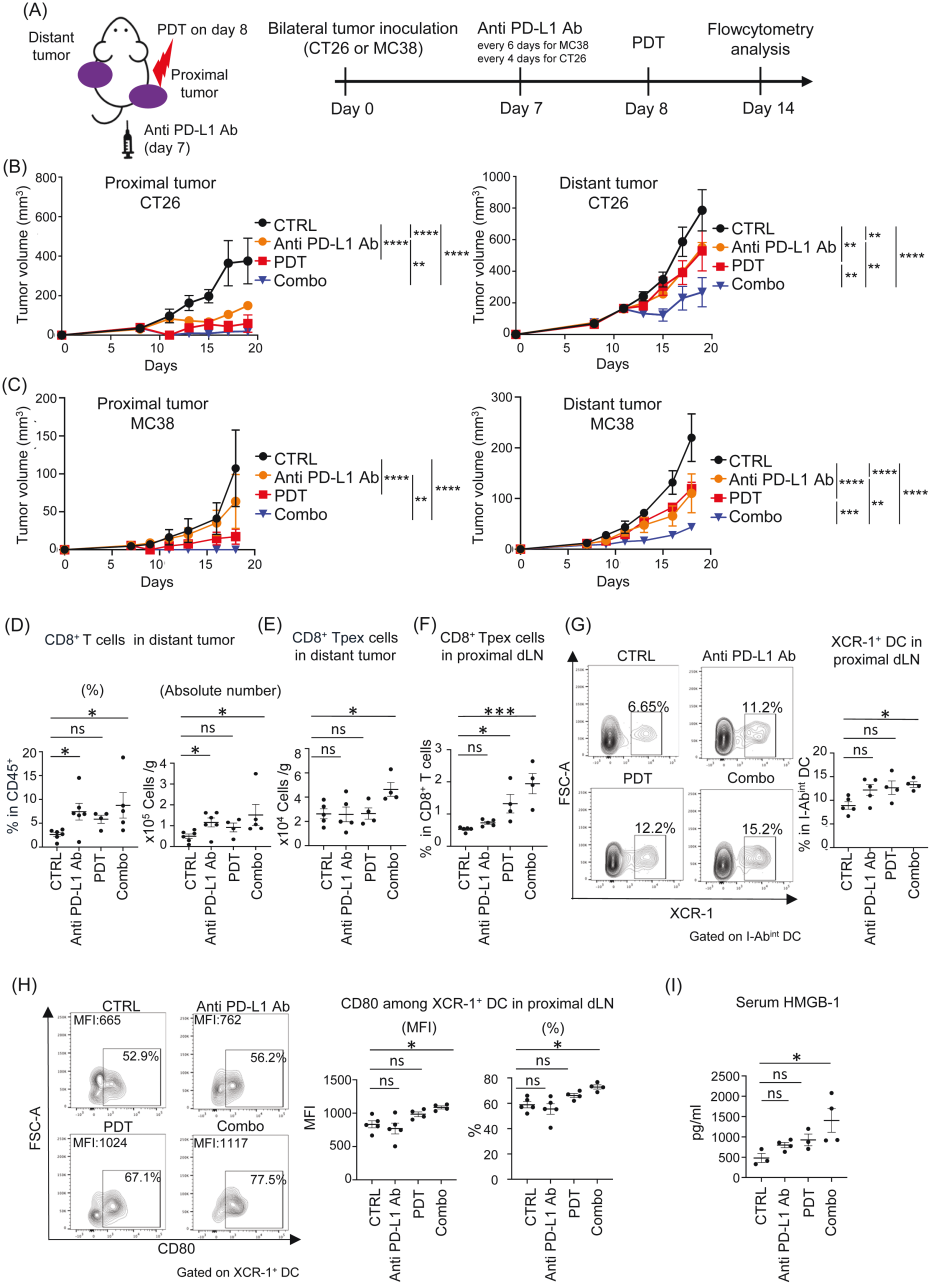
The combination therapy of Tal-PDT and anti-PD-L1 Ab induces a synergistic abscopal effect, characterized by increased Tpex cells in the laser-untreated distant tumor and activation of the XCR-1^+^ DCs in the proximal dLN. (A) Illustration of the experiment. (B and C) CT26 (B) and MC38 (C) tumor growth of the proximal tumor (left panel) and the distant tumor (right panel) (*n* = 3-4). (D-H) In the MC38 tumor model, mice were sacrificed on Day 14 and flow cytometry analyses of the distant tumor (D and E) and the proximal dLN (F–H) were performed. The percentage of tumor-infiltrating CD8^+^ T cells among total CD45^+^ cells (left panel) and their absolute counts (right panel) are shown (D). The absolute number of CD8^+^ Tpex cells is defined as (CD44^+^, PD-1^int^, TCF-1^+^, Tim-3^−^) in the distant tumor (E). The percentage of the Tpex cells in the proximal dLN (F). The percentage of XCR-1 expression among DCs (CD11c^+^, I-Ab^int^) in the proximal dLN (G). CD80 expression (MFI; left panel, %; right panel) in XCR-1^+^ DCs in the proximal dLN (H). (I) Serum HMBG-1 levels were measured by ELISA 48 hours after PDT in the MC38 tumor model. Representative flow cytometry staining results are shown in the left panels (G and H). Gating strategy of Tpex and DC is shown in Supplementary Fig. 3. Combo; the combination therapy of PDT and anti PD-L1 antibody. The data are expressed as means ± SEM. Statistical analyses were performed using two-way ANOVA followed by Tukey’s multiple comparisons test (Day 19; B and Day18; C), Kruskal–Wallis test followed by Dunn’s multiple comparisons test (D) and one-way ANOVA followed by Dunnett’s multiple comparisons test (E-I). The data are representative of two independent experiments. *P < .05, **P < .01, ***P < .001 ****P < .0001. The data are representative of two independent experiments.

As the abscopal effect is mediated by immunity, we tested whether the abscopal effect of Tal-PDT enhances the efficacy of PD-1 blockade therapy. In bilateral cancer models using MC38 or CT26 cells, which express PD-L1 (Supplementary Fig. 1A), mice were treated with anti-PD-L1 mAb on Day 7, followed by Tal-PDT on Day 8 (Fig. 3A). In both CT26-harboring BALB/c mice and MC38-harboring C57BL/6 mice, synergistic antitumor effects were observed in both proximal and distant tumors (Fig. 3B and C). Subsequently, we conducted immunological analysis using flow cytometry on day 14 in the MC38-tumor-bearing C57BL/6 mouse model, following the completion of two doses of anti-PD-L1 mAb. We confirmed expression of the target of the antibody, PD-L1, on both tumor cells and DCs in the dLN (Supplementary Fig. 1B and C). Although the enhancement of CD8^+^ T-cell infiltration in the distant tumor tissue was comparable between the combination therapy and single therapies at this time point (Fig. 3D), the number of the CD8^+^ Tpex cells was elevated only in the combination therapy group (Fig. 3E). This might account for the observed synergistic antitumor effects (Fig. 3C). Since the percentage of Tpex cells increased in the proximal dLN in both the PDT and combination therapy groups (Fig. 3F), we examined XCR-1^+^ DCs in the proximal dLN. XCR-1^+^ DCs were previously reported to be master cross-presenting DCs (25) and to have the ability to expand the Tpex pool (26–28). We found that the combination therapy increased the frequency of XCR-1^+^ DCs (Fig. 3G), which exhibited a more mature phenotype, as evident by elevated CD80 expression (Fig. 3H). Because we observed even Tal-PDT single therapy could slightly increase and activate DCs though the results were not statistically significant (Fig. 3G and H), we further investigated the effect of Tal-PDT on DC function to stimulate CD8⁺ T cells. DCs were collected from the proximal dLNs following PDT treatment and co-cultured with CFSE-prelabeled naïve OT-1 CD8⁺ T cells with the OVA peptide (Supplementary Fig. 2A). DCs from PDT-treated mice showed more enhanced ability to induce OT-1 CD8⁺ T cell proliferation compared to those from the control mice (Supplementary Fig. 2B), suggesting Tal-PDT could enhance DC function in the dLN. One of the DAMPS, HMGB-1, stimulates DCs upon its release from dying cells (29). We detected a higher concentration of HMGB-1 in the sera of the combination therapy group two days after PDT (Fig. 3I), which likely contributed to the activation of XCR-1^+^ DCs in the proximal dLN.

### The dLN of laser-treated tumors is essential for the abscopal effect of PDT

**Figure 4.**
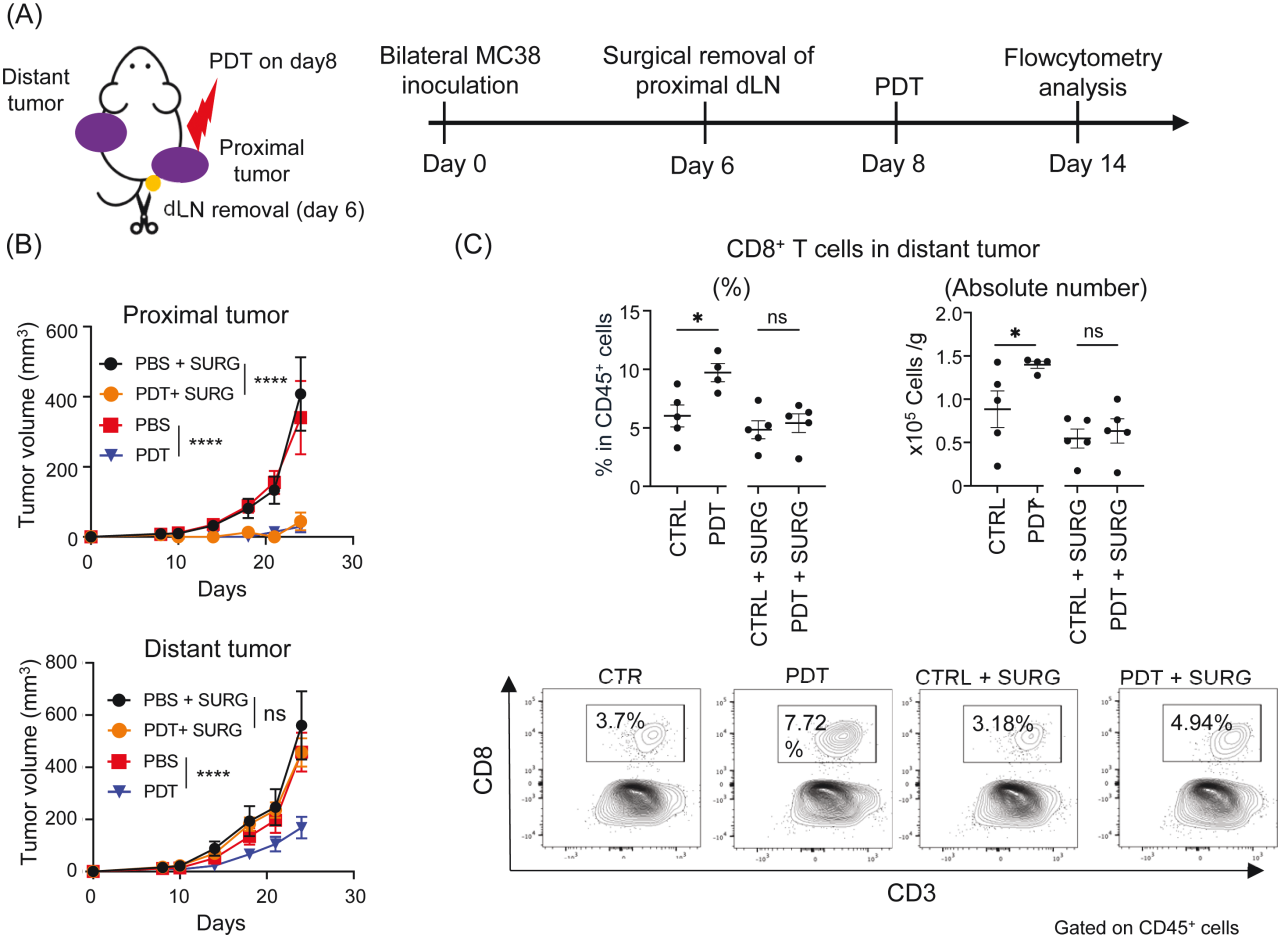
The dLN of the laser-treated tumor is essential for the effective abscopal effect induced by Tal-PDT. (A) Illustration of the experiment. (B) Tumor growth of the proximal tumor (top panel) and the distant tumor (bottom panel) is shown (*n* = 4). (C) The percentage (left top panel) and absolute count (right top panel) of CD8^+^ T cells infiltration in distant tumor, with or without the proximal dLN, are shown. Representative flow cytometry staining results are shown in the bottom panel. Data are expressed as means ± SEM. Statistical analyses were performed using two-way ANOVA followed by Tukey’s multiple comparisons test (Day 24; B) and Mann Whitney test (C). *P < .05, ****P < .0001. ns; not significant. The data are representative of two independent experiments.

Tumor dLNs play a crucial role in the immune response against tumors. Since treating tumors with Tal-PDT generated XCR-1^+^ DCs and Tpex cells in the proximal dLNs (Fig. 3F-H), we investigated whether these dLNs contribute to the abscopal effect. To do this, in a bilateral tumor model, we surgically removed the dLN from the proximal tumor two days before Tal-PDT treatment. Then, we evaluated the growth of both proximal and distant tumors (Fig. 4A). We found that removing the dLN from the proximal tumor did not affect the antitumor activity of Tal-PDT on the proximal tumor but completely abolished the abscopal effect on distant tumor growth (Fig. 4B). Moreover, in the group with the dLN removal, infiltration of CD8^+^ T cells into the distant tumor was also substantially reduced (Fig. 4C). These data demonstrate that the dLN of the PDT-treated tumor (proximal tumor) plays a crucial role in mediating the abscopal effect.

### Talaporfin is selectively incorporated into tumor cells rather than T cells

**Figure 5.**
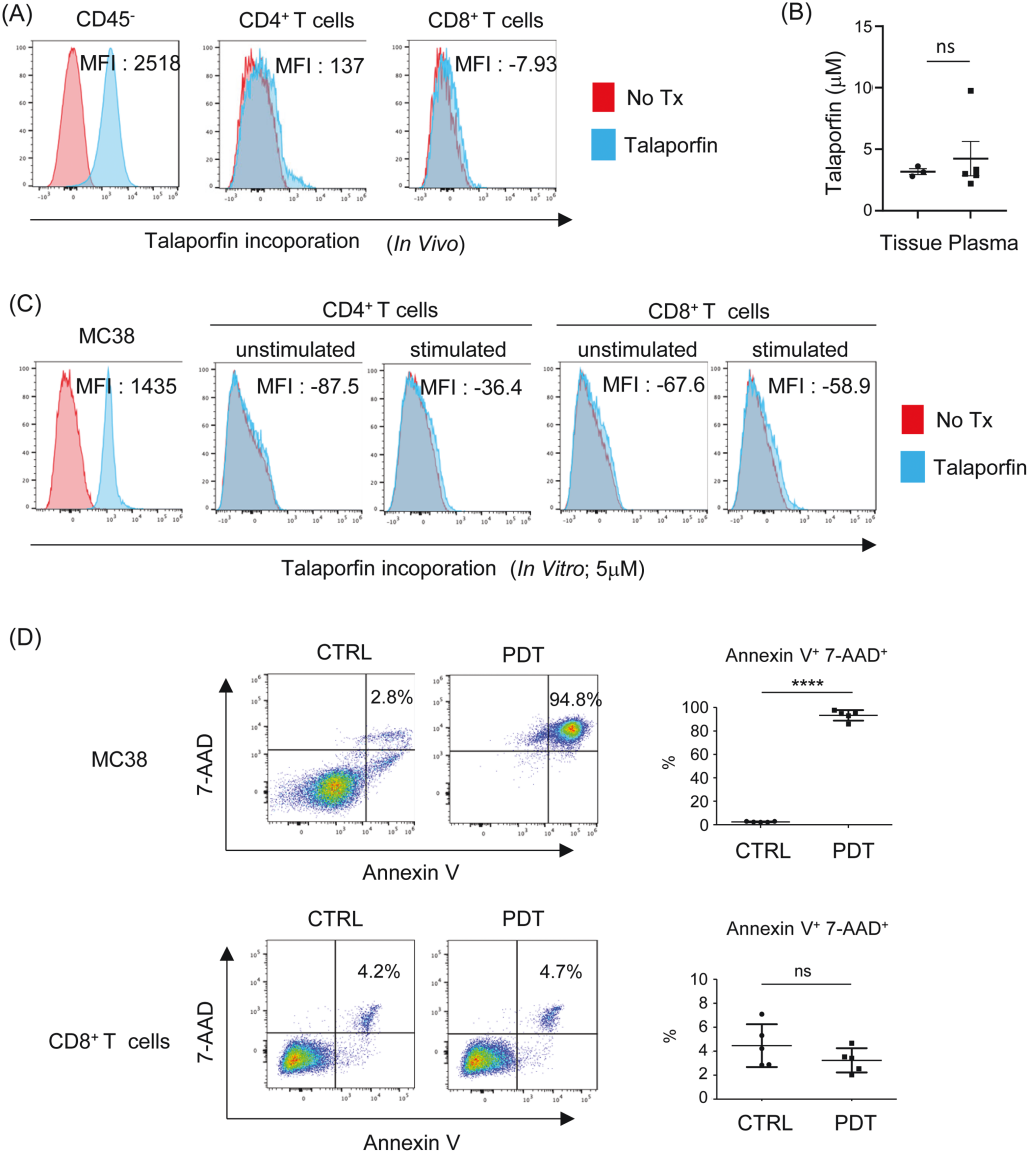
Talaporfin is preferentially incorporated into tumor cells rather than into T cells. (A and B) MC38 tumor tissues were resected 2 hours after talaporfin administration and digested. Talaporfin incorporation was measured in T cells and non-immune cells, including tumor cells (CD45^−^), using flow cytometry (A). Talaporfin concentration in sera and tumor tissues measured by LC–MS (B). (C) MC38 cells or murine splenic T cells (both unstimulated and those stimulated with anti-CD3 antibody for 3 days) were incubated with 5 µM of talaporfin for 2 hours. Talaporfin incorporation was measured by flow cytometry. (D) After a 2-hour incubation with talaporfin, the laser was applied to the cells. Cell death was assessed by Annexin-V/7-AAD staining 24 hours after laser irradiation. Data are expressed as means ± SEM. Statistical analyses were performed using *t*-test. ****P < .0001. ns; not significant. The data are representative of 2–3 independent experiments.

Given that Tal-PDT successfully induced Tpex cells and increased CD8^+^ T-cell infiltration in tumors, we hypothesized that Tal-PDT has reduced cytotoxicity to T cells. To test this hypothesis, we compared the sensitivity of tumor cells and T cells to cell death induced by Tal-PDT. There was minimal talaporfin incorporation in tumor-infiltrating CD4^+^ or CD8^+^ T cells *in vivo*, compared to its higher presence in nonimmune CD45^-^ cells, which were predominantly tumor cells (Fig. 5A). To compare the PDT sensitivity of MC38 and murine T cells *in vitro*, we measured the physiological concentration of talaporfin in plasma and tumor tissue 2 hours after talaporfin injection. This timing corresponds to when the laser is emitted in both our mouse models and clinical settings (Fig. 5B). In the presence of talaporfin at a physiological concentration of 5 uM, murine T cells displayed resistance to Tal-PDT-induced cell death, likely because of minimal incorporation of talaporfin *in vitro* (Fig. 5C and D). These results indicate that Tal-PDT did not affect tumor-infiltrating T cells in the laser-treated tumor tissue, while it strongly induced cell death in tumor cells. This explains the presence of effector CD8^+^ T cells in the tumors during Tal-PDT treatment.

## Discussion

We demonstrated that combining Tal-PDT with PD-1 inhibitors synergistically enhanced tumor-specific immunity, leading to the abscopal effect, as previously reported (18, 30). In this study, we have investigated the mechanism by which Tal-PDT induces the abscopal effect. On the basis of our results, we propose the following mechanism for the observed abscopal effect. (i) Tal-PDT-induced ferroptosis of cancer cells was associated with the induction of proliferative CD8^+^ Tpex cells possibly through the XCR-1^+^ DC maturation in the proximal dLN. (ii) Taraporfin was preferentially incorporated into cancer cells at the tumor site, thereby protecting tumor-infiltrating CD8^+^ T cells from the cytotoxic effects of Tal-PDT during therapy. Collectively, this would generate long-lived CD8^+^ T cells and enhance systemic antitumor immunity.

Recently, the importance of dNLs in ICB cancer immunotherapy has become more evident (27, 28, 31, 32). Studies have shown that XCR-1^+^ cDC1s increase in lymphoid organs following ICB treatment (33). Additionally, XCR-1^+^ DCs are essential for inducing Tpex cells in the dLN (26–28). After migrating to tumor tissues, Tpex cells differentiate into effector T cells, replacing the pre-existing terminally exhausted T cells in a process known as clonal replacement (34). Given the synergistic abscopal effect observed with combination therapy, Tal-PDT may accelerate clonal replacement by ICB therapy in distal tumors. Our data show that inhibition of ferroptosis or removing the proximal dLN reduced the number of CD8^+^ T cells infiltrating the distal tumor. This indicates that ferroptosis of cancer cells enhanced the generation of XCR-1^+^ cDC1s and stimulated Tpex cells in the proximal dLN, which then circulated systemically to the opposite tumor site. The increased CD80 on the surface of XCR-1^+^ DCs by Tal-PDT in the dLN might be also important because sustained CD28 stimulation is reported to be essential for maintaining the Tpex pool (35). Therefore, the generation of XCR-1^+^ cDC1s and the increased expression of CD80 by Tal-PDT in the dLN are likely key to the abscopal effect.

We demonstrated that the ferroptosis of cancer cells induced by Tal-PDT is crucial for the abscopal effect. This result suggests that ferroptosis of cancer cells is an immunogenic form of cell death, which enhances antitumor immunity. Nevertheless, the impact of tumor ferroptosis on immunity is controversial. Several studies reported that ferroptosis inhibits immune activity by secreting prostaglandin E2 (PGE2) and HMGB-1 and, thereby, activating bone marrow-derived suppressor cells (MDSCs) and Tregs (36, 37). Moreover, ferroptosis increases the PD-L1 expression on the surface of cancer cells, suppressing immunity. This effect can be reverted by adding the PD-1/PD-L1 blocking antibodies to the cancer cells (36). The positive impact on anti-tumor immunity suggests that ferroptosis-derived ATP and HMGB-1 activate bone marrow-derived DCs, which aligns with our results. Notably, cancer cells undergoing ferroptosis release sufficient ATP and HMGB-1 to activate DCs within one to three hours, but not after 24 hours (29). Given that Tal-PDT induces cell death immediately (38), it may be effective for activating cDC1s. Recently, it has been reported that cDC1s are activated through the DNA sensing pathway involving cGAS/STING in both tumor-bearing mice and cancer patients (39). Cytosolic DNA released by Tal-PDT-induced ferroptosis would be detected by the STING signaling pathway and may contribute to the cDC1 induction in our model. Further studies are necessary to reveal the detailed mechanism by which Tal-PDT induces XCR-1^+^ cDC1s.

Another possible mechanism for the abscopal effect induced by Tal-PDT is its differential toxicity of Tal-PDT to tumor cells compared to tumor-infiltrating T cells. Although it has been reported that talaporfin is preferentially incorporated into cancer cells rather than PBMCs *in vitro* (40), we are the first to demonstrate that talaporfin uptake is high in cancer cells and very rare in tumor-infiltrating T cells *in vivo*. This difference in uptake is crucial because minimizing Tal-PDT toxicity to tumor-infiltrating Tpex cells can help preserve these cells, allowing for the production of fresh effector T cells, which are essential for the abscopal effect. Although the mechanism of talaporfin incorporation into cells is not fully understood, it is possible that its uptake is mediated by lyposomal endocytosis, which is regulated by intracellular ATP levels (41). Since ATP production is higher in cancer cells than in T cells, talaporfin could be selectively accumulated in tumor cells (41).

In summary, we demonstrated that Tal-PDT enhanced systemic antitumor immunity triggered by ICB. Specifically, Tal-PDT-derived ferroptosis of cancer cells induced XCR-1^+^ cDC1s and Tpex cells, which may give rise to the effector T cells *in vivo*. These data underscore the immunological impacts of Tal-PDT and provide insights into the potential for combing this therapy with ICB for clinical application.

## Supplementary Material

dxaf003_suppl_Supplementary_Tables

dxaf003_suppl_Supplementary_Figures
