# Supplementary material for: Beneficial effects on T cells by photodynamic therapy with talaporfin enhance cancer immunotherapy
Source: Int Immunol. 2025 Jan 22;37(6):313–24. doi: 10.1093/intimm/dxaf003 (PMC12096158; doi:10.1093/intimm/dxaf003)
Supplement: dxaf003_suppl_Supplementary_Tables [file dxaf003_suppl_supplementary_tables.docx]

**Supplementary Table S1**

The list of antibodies used in this study

| **Antibody** | **Manufacture** | **Clone** | **Identifier #** | **Dilution** |
| --- | --- | --- | --- | --- |
| CD3 BUV805 | BD Biosciences | 145-2C11 | 749276 | 1:200 |
| CD8a APC | Biolegend | 53-6.7 | 17-0081-82 | 1:200 |
| CD8 FITC | MBL | KT15 | K0227-4 | 1:200 |
| CD8a BUV737 | BD Biosciences | 53-6.7 | 612759 | 1:200 |
| CD8a AF488 | BD Biosciences | 53-6.7 | 557668 | 1:100 |
| CD44 BUV661 | BD Biosciences | IM7 | 741471 | 1:200 |
| CD62L PE/CY7 | Biolegend | MEL-14 | 104418 | 1:200 |
| PD-1 BV421 | Biolegend | RMP1-30 | 135221 | 1:200 |
| Tim-3 BB700 | BD Biosciences | 5D12/TIM-3 | 747619 | 1:200 |
| TCF1/TCF7 AF488 | Cell Signaling | C63D9 | 6444S | 1:100 |
| CD69 APC | Biolegend | H1.2F3 | 104514 | 1:200 |
| CD45 BUV395 | BD Biosciences | 30-F11 | 564279 | 1:200 |
| F4/80 BUV805 | BD Biosciences | T45-2342 | 749282 | 1:200 |
| Gr-1 PerCp/Cy5.5 | Biolegend | RB6-8C5 | 108427 | 1:200 |
| CD11c BV421 | Biolegend | N418 | 117330 | 1:200 |
| I-A[b] FITC | BD Biosciences | 2025/09/17 | 553605 | 1:200 |
| XCR1 APC | Biolegend | ZET | 148206 | 1:200 |
| CD80 PE | Invitrogen | 16-10A1 | 104708 | 1:200 |
| CD3 BUV737 | BD Biosciences | 17A2 | 612803 | 1:200 |
| CD16/CD32 | BD Biosciences | 2.4G2 | 553142 | 1:100 |
| CD274 (PD-L1, B7-H1). PE | BioLegend | 10F.9G2 | 124313 | 1:200 |
| CD8a. PerCp/Cy5.5 | Biolegend | 53-6.7 | 100733 | 1:200 |

**Supplementary Table S2**

The list of reagents used in this study

| **Reagent** | **Manufacture** | **Identifier #** |
| --- | --- | --- |
| Foxp3/Transcription Factor Fixation/Permeabilization Concentrate and Diluent | Thermo Fisher Scientific, Invitrogen™ | 00-5521-00 |
| COLLAGENASE TYPE 4 | Worthington Biochemical Corporation | 9001-12-1 |
| Liproxstatin-1 | Sigma‒Aldrich | SML1414-5MG |
| BODIPY™ 581/591 C11 (Lipid Peroxidation Sensor) | Thermo Fisher Scientific, Invitrogen™ | D3861 |
| Zombie NIR Fixable Viability Kit | Biolegend | 423105 |
| UltraComp eBeads Compensation Beads | Thermo Fisher Scientific, Invitrogen | 01-2222-42 |
| T-Select H-2Kb Negative (SIY) Tetramer-SIYRYYGL-PE | MBL international corporation | TS-M008-1 |
| T-Select H-2K b β-galactosidase Tetramer-DAPIYTNV-PE MBL, | MBL international corporation | TS-M501-1 |
| CultureSure® DMSO | Fujifilm Wako Pure Chemical Industries | 031-24051 |
| Puromycin Dihydrochloride | Thermo Fisher Scientific, Gibco™ | A1113803 |
| Trypsin-EDTA (0.25%),  phenol red | Thermo Fisher Scientific, Gibco™ | 25200056 |
| CD8a+ T-Cell Isolation Kit, mouse, MACS cell separation | Miltenyi Biotec | 130-104-075 |
| CD11c MicroBeads,  UltraPure mouse Kit | Miltenyi Biotec | 130-125-835 |
| CellTrace™ CFSE Cell Proliferation Kit | Thermo Fisher Scientific,  Invitrogen™ | C34570 |
| H-2Kb OVA peptide | MBL international corporation | TS-5001-P |
| IL-2 (PROLEUKIN^®^) | Novartis | - |
| Dynabeads® Mouse T-Activator CD3/CD28 | Thermo Fisher Scientific, Gibco™ | 11456D |
| Apotracker™ Green | Biolegend | 427402 |
| Brilliant Stain Buffer | Thermo Fisher Scientific, Invitrogen™ | 00-4409-75 |
| Camptothecin | Fujifilm Wako Pure Chemical Industries | 1100/25 |
| Mouse HMG1/HMGB1 (Sandwich ELISA) ELISA Kit | LifeSpan Biosciences | LS-F23080-1 |
| InVivoMAb rat IgG2a isotype control | BioXcell | BE0089 |
| FITC Annexin V Apoptosis Detection Kit with 7-AAD | Biolegend | 640922 |
| CountBright Absolute Counting Beads | Thermo Fisher Scientific,  Invitrogen™ | C36950 |
| Mouse IFN-gamma Recombinant Protein, | Thermo Fisher Scientific,  PeproTech® | 315-05-100UG |
