# Supplementary material for: Beneficial effects on T cells by photodynamic therapy with talaporfin enhance cancer immunotherapy
Source: Int Immunol. 2025 Jan 22;37(6):313–24. doi: 10.1093/intimm/dxaf003 (PMC12096158; doi:10.1093/intimm/dxaf003)
Supplement: dxaf003_suppl_Supplementary_Figures [file dxaf003_suppl_supplementary_figures.zip › II_Revise_Figure legend for supplementary figures/II_Revise_Figure legend for supplementary figures.docx]

**Supplementary Figure 1: PD-L1 expression in CD45⁻ cells and CD11c⁺ DCs in dLN (A)** PD-L1 expression on MC38 and CT26. The cells were treated with or without IFN-γ for 24h *in vitro* and PD-L1 expression was measured by flow cytometer. Each ΔMFI (calculated by subtracting the MFI of the unstained sample) is shown. **(B)** PD-L1 expression on CD45^-^ cells, which are mainly composed of MC38 cells, in MC38 tumor tissues was measured on day14 by flow cytometry. **(C)** PD-L1 expression on DC in the dLN was measured on day14 by flow cytometry. Data are expressed as means ± SEM. Statistical analyses were performed using one-way ANOVA followed by Dunnett's multiple comparisons test. ****p < 0.0001, ns; not significant

**Supplementary Figure 2: PDT enhances the priming function of DCs in dLN**

**(A)** An illustration of the experiment. DCs, which were collected from dLN of MC38-bearing mice on day 14, and CFSE-labeled OT-1 CD8⁺ T cells, which are obtained from the spleen of an OT-1 mouse, were co-cultured (DC:T cells=1:5) for 3 days with OVA peptide (0.5 µg/ml) and IL-2 (20 IU/ml). The OT-1 T cell proliferation was evaluated by CFSE dilution using flow cytometer. **(B)** The number and the percentage of proliferating OT-1 CD8⁺ T cells are shown (right panel). Representative flow cytometer histograms are shown (left panel). Data are expressed as means ± SEM. Statistical analyses were performed using unpaired Student's t-test. *p < 0.05, **p < 0.01. The data are representative of 2 independent experiments.

**Supplementary Figure 3: Gating strategy of DC and T pex in dLN.**

**(A)** Gating strategy of DC **(B)** Gating strategy of Tpex in dLN **(C)** Gating strategy of Tpex in TILs
