# Supplementary material for: Beneficial effects on T cells by photodynamic therapy with talaporfin enhance cancer immunotherapy
Source: Int Immunol. 2025 Jan 22;37(6):313–24. doi: 10.1093/intimm/dxaf003 (PMC12096158; doi:10.1093/intimm/dxaf003)
Supplement: dxaf003_suppl_Supplementary_Figures [file dxaf003_suppl_supplementary_figures.zip › II_Revise_Figure legend for supplementary figures/II_Revise_Supple Figure.pdf]

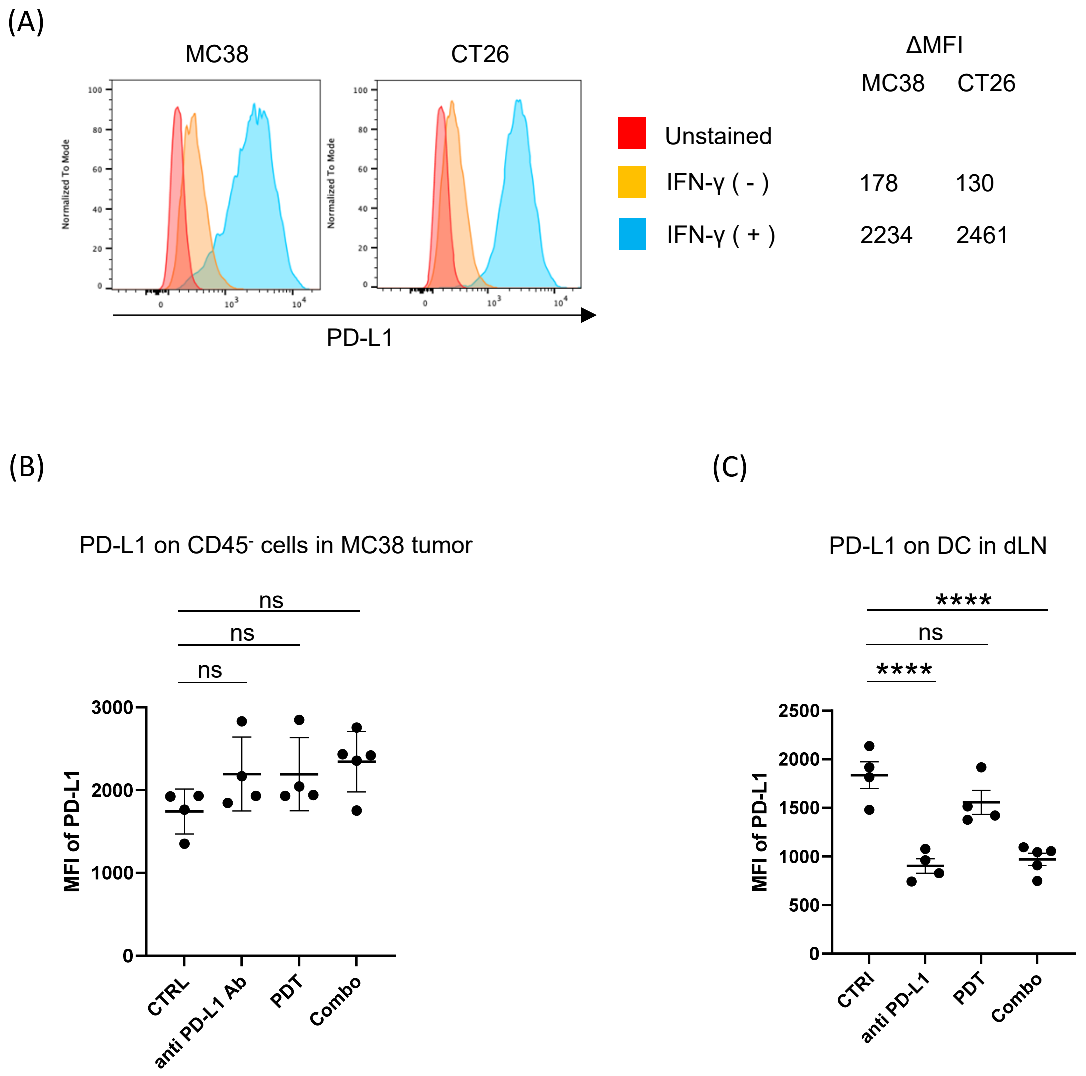

Supplementary Figure 1

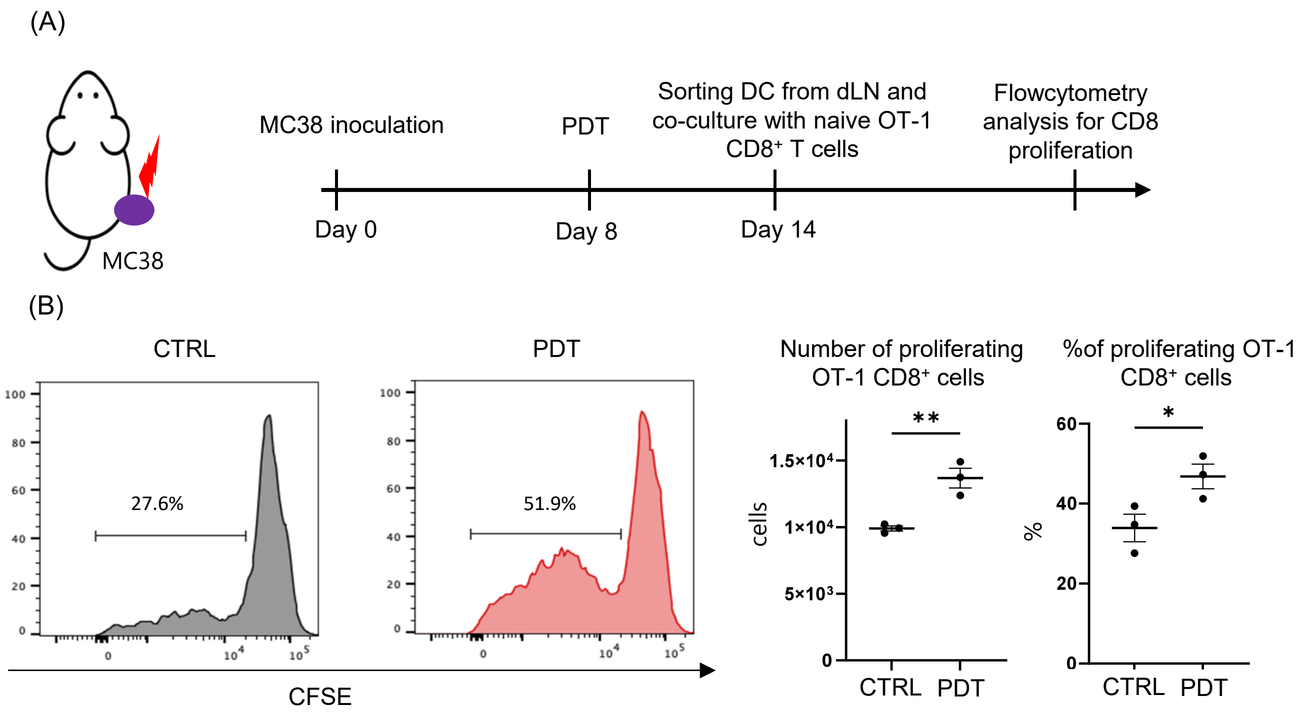

Supplementary Figure 2

(A) Gating strategy of DC :

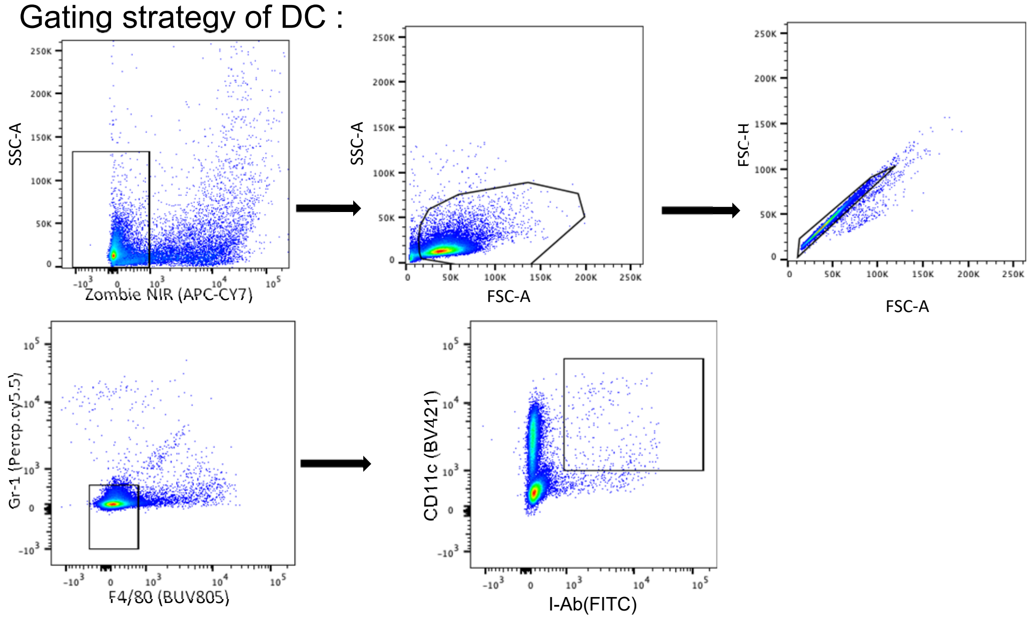

(B) Gating strategy of Tpex in dLN :

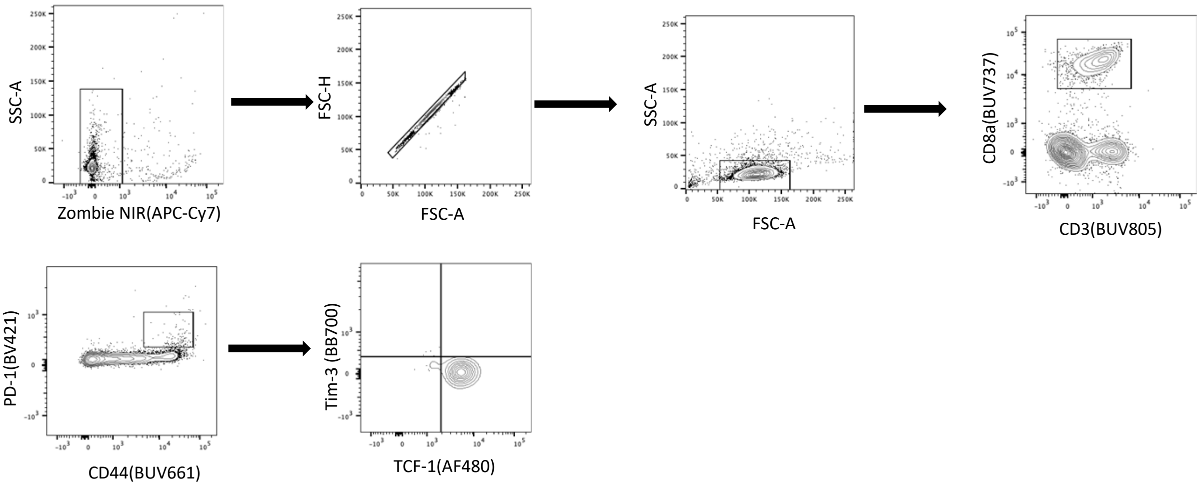

(C) Gating strategy of Tpex in TILs :

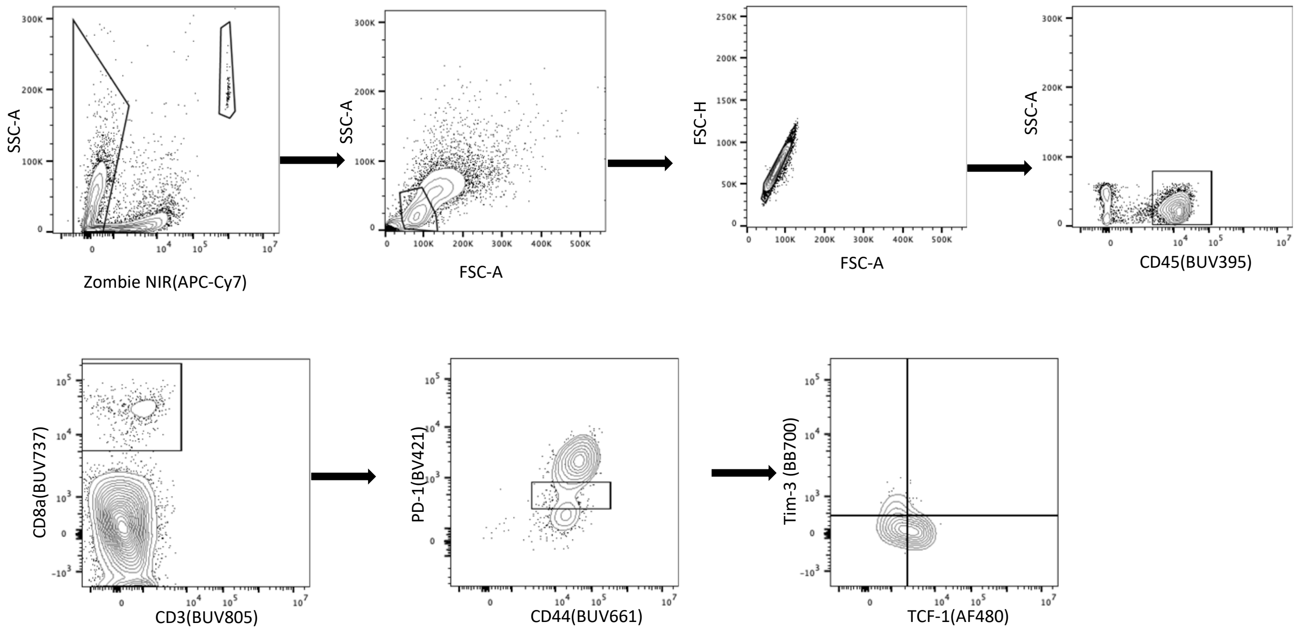

Supplementary Figure 3
